# Prevalence, Causes, and Risk Factors of Visual Impairment: Evidence from Duhknah, a Rural Community in Saudi Arabia

**DOI:** 10.3390/healthcare13151927

**Published:** 2025-08-07

**Authors:** Sulaiman Aldakhil

**Affiliations:** Department of Optometry, College of Applied Medical Sciences, Qassim University, Buraydah 51452, Saudi Arabia; s.dakhil@qu.edu.sa; Tel.: +966-5558-78739

**Keywords:** visual impairment, rural community, quality of life, public health, risk factors, refractive errors, myopia

## Abstract

**Background:** Visual impairment (VI) continues to be a significant global public health concern, especially in underserved rural communities. **Objectives**: This study aims to assess the prevalence of VI and refractive errors, as well as to identify the causes and risk factors associated with VI in Duhknah, a rural area in Qassim Province, Saudi Arabia. **Methods**: This cross-sectional study, conducted in May 2024, included 929 participants aged 6–90 years from Duhknah, a rural area in Qassim Province, Saudi Arabia. Refractive errors (REs) were measured using a non-cycloplegic autorefractometer. Anterior and posterior eye examinations were performed using slit lamp biomicroscopy, direct ophthalmoscopy, and 90 D fundus biomicroscopy. VI was classified based on the International Classification of Diseases 11th revision (ICD-11), 2018. **Results**: The findings revealed that 671 (72.2%) participants had never undergone an eye examination. The overall prevalence of presenting VI was 370 (39.8%), comprising 21.6% with mild VI, 11.0% moderate, 4.1% severe, and 3.1% classified as blind. The prevalence of hyperopia, myopia, and astigmatism was 20.6%, 36.9%, and 13.2%, respectively. Uncorrected REs were the most common cause of VI (81.4%), followed by amblyopia (13.5%) and cataracts (3.2%). Regression analysis showed that women had 1.58 times higher odds of VI (*p* = 0.001). Participants with eye examinations for one year or more had 3.64 times higher odds (*p* < 0.001). Additionally, the risk of VI was significantly lower among older participants (ages 18–90) compared to younger ones (ages 6–17), (*p* < 0.001). **Conclusions**: This study found most participants had never had an eye exam, and VI was highly prevalent in the rural community. These findings underscore the need to strengthen primary eye care in rural Saudi Arabia. Regular vision screening, particularly for children, and better access to refractive services could significantly reduce VI and support the goals of Saudi Vision 2030.

## 1. Introduction

Primary eye care is an essential component of the healthcare system, playing a critical role in early detection, management, and prevention of eye conditions [1]. Limited access to quality eye care services can result in high prevalence of VI, which pose significant long-term threats to vision and overall well-being [2]. Such impairments can profoundly impact an individual’s quality of life, limiting daily activities, reducing independence, and increasing the risk of social isolation [1,3]. Furthermore, untreated VI place a substantial burden on healthcare systems and economies, leading to increased healthcare costs and decreased productivity [1,2,3,4].

Most causes of VI are preventable with timely intervention and appropriate management. In rural communities of Saudi Arabia, the reported prevalence of VI ranges between 13.9% and 32.1% according to previous studies [5,6,7], underscoring a substantial public health concern. The primary causes consistently identified across these studies include uncorrected refractive errors, cataracts, amblyopia, and glaucoma, all of which can lead to blindness if not promptly diagnosed and treated [5,6,7]. Fortunately, these conditions are largely avoidable and can be effectively managed through accessible, affordable, and well-integrated primary eye care services.

Notably, the highest prevalence rates of VI have been reported in studies conducted in rural areas, where access to primary care services tends to be limited [5,7]. This disparity emphasizes the critical importance of assessing visual health in underserved regions, as such data reflects the availability and quality of eye care services within these communities. It is important to conduct more studies in rural areas to better understand the gaps in healthcare accessibility and implement sustainable interventions. Considering this, the current study aims to assess the prevalence of VI and refractive errors, as well as to identify the causes and risk factors associated with VI in Duhknah, a rural area in Qassim Province, Saudi Arabia.

## 2. Materials and Methods

### 2.1. Study Design and Participants

The researcher conducted this descriptive cross-sectional study in May 2024 in Duhknah, a rural community in the Qassim Province of Saudi Arabia. In the community eye health campaign, a total of 929 participants aged between 6 and 90 years were examined. Qassim University organizes this medical campaign annually, providing healthcare services, including eye care examinations.

### 2.2. Inclusion and Exclusion Criteria

Participants aged 6–90 years who attended the medical campaign and consented to participate were included. Children without parental consent or who did not agree to participate were excluded.

### 2.3. Study Sample

The study sample was calculated based on the following. According to the 2022 census, the total population of Duhknah is 6354. Assuming a prevalence of VI 24.5%, based on the estimated prevalence among a rural community in Saudi Arabia [5]. With a 95% confidence level and a maximum acceptable margin of error of 3.8%, the initial estimated sample size was 619, calculated using the formula below [8]. After accounting for a design effect of 1.5 to adjust for sampling variability, the final sample size was increased to 929.

n = (z^2^ pq)/d^2^ = (1.96^2^ × 0.245 × 0.95)/0.038^2^ = 619.2 => 619 × 1.5 = 929.

Considering a nonresponse rate of 10% (equivalent to 93 individuals), the target sample size was increased to 1022 participants.

### 2.4. Ethical Consideration

The study was approved by the Qassim University Health Research Ethics Committee (Approval Number: 19-07-06) and conducted in accordance with the Declaration of Helsinki. Data confidentiality was strictly maintained, and no personal information was disclosed. All participants who underwent eye examinations were included in the study, and written informed consent was obtained from each participant.

### 2.5. Clinical Collection Procedures

All subjects underwent comprehensive eye examinations, including visual acuity (VA), ocular health assessment of their anterior and posterior eye segments, as well as non-cycloplegic refraction and subjective refraction, were performed. Experienced eye care practitioners from Qassim University, including ophthalmologists and optometrists, performed these examinations. In this study, the International Classification of Diseases 11th revision, 2018 (ICD-11) was used to classify the presenting VI. In this study, mild visual impairment is defined as visual acuity worse than 6/12 but equal to or better than 6/18, moderate visual impairment refers to visual acuity worse than 6/18 but equal to or better than 6/60, severe visual impairment is characterized by visual acuity worse than 6/60 but equal to or better than 3/60, and blindness is defined as visual acuity worse than 3/60 [9]. All examinations were collected in one week and the data included age, date of last eye examination, main complaint, VA, and RE. The measurements of RE were obtained objectively using a non-cycloplegic autorefractometer (NIDEK autorefractor (RK-310)). Myopia was defined as RE with at least −0.5 diopters (D) in one or both eyes, hyperopia as RE with at least +1.00 D in one or both eyes, and astigmatism as cylindrical refraction at 0.75 D or more. Slit lamp biomicroscopy was used to assess the anterior eye examination, and direct ophthalmoscope and 90 D fundus biomicroscopy were also performed to evaluate the posterior segment of the eye.

### 2.6. Data Analysis

The data were initially entered into Microsoft Excel, where ocular examination measurements were reviewed and checked for any missing values. Subsequently, statistical analyses were conducted using SPSS statistical software (Version 24). Percentages and frequencies were used to summarize categorical variables, while the findings related to refractive error, visual impairment, and its subgroups were descriptively analyzed using cross-tabulation. MedCalc statistical software, version 23.1.7, was used to analyze the prevalence of visual impairment and refractive error by age group and gender. Risk factors associated with visual impairment including age, gender, date of last eye examination, and uncorrected refractive errors were analyzed using logistic regression, with adjusted odds ratios and 95% confidence intervals calculated to assess their significance. Statistical significance was set in all analyses at *p*-value of <0.05.

## 3. Results

### 3.1. Demographic Characteristics

A total of 1022 subjects were invited to participate in the community study. Of these, 929 agreed to take part, yielding a participation rate of 90.9%. Participants ranged in age from 6 to 90 years, with a mean age of 21.2 ± 17.6 years. The study comprised 487 males (52.4%) and 442 females (47.6%). Most participants, 671 (72.2%), reported never having undergone an eye examination before, while only 121 (13.0%) reported using eyeglasses. VI was significantly associated with increased age and female gender (*p* = 0.0001), as detailed in Table 1. The Chi-square test was used in Table 1 to examine associations between VI and categorical variables such as age group, gender, last eye examination date, and eyeglass usage.

### 3.2. Main Ocular Complaints

Among the participants, 69% visited for a routine check-up, while 20.4% reported blurry vision, 3.5% had dry eyes, 2.7% experienced headaches, 1.4% reported itching, 2.0% had redness, and only 1.0% experienced tearing.

### 3.3. Prevalence of Presenting Visual Impairment Among the Study Participants

In this study, the overall prevalence of presenting VI among participants was 39.8%, as shown in Table 1. This included mild VI at 21.6%, moderate VI at 11.0%, severe VI at 4.1%, and blindness at 3.1%. These findings are detailed by age group and gender in Table 2. The Chi-square test was used in Table 2 to assess the association between the prevalence of presenting VI and categorical variables such as age group and gender.

### 3.4. Prevalence of Refractive Error

The prevalence of RE in one or both eyes, classified by age and gender, is presented in Table 3. The results showed that the overall prevalence of RE among participants was 70.7% (657 individuals; 95% CI, 65.4–76.3). A statistically significant association was found between the prevalence of RE and both age (*p* = 0.000) and gender (*p* = 0.025), respectively.

Hyperopia, myopia, and astigmatism were observed in 20.6% (191 individuals; 95% CI, 17.8–23.7), 36.9% (343 individuals; 95% CI, 33.1–41.0), and 13.2% (123 individuals; 95% CI, 11.0–15.8) of subjects, respectively. Myopia was more common in the age group of 18–39 (60.2%; 118 individuals; 95% CI, 49.8–72.1) and among males (37.6%; 183 individuals; 95% CI, 32.3–43.4). In contrast, hyperopia was found in individuals aged 60 and above (47.7%; 21 individuals; 95% CI, 29.5–72.9) and among females (22.2%; 98 individuals; 95% CI, 18.0–27.0). The Chi-square test was used in Table 3 to assess the association between the prevalence of refractive error and categorical variables such as age group and gender.

### 3.5. Risk Factors for Developing Visual Impairment Among the Participants

Regression analysis showed that the likelihood of VI decreased with age. Specifically, individuals aged 18–90 had lower odds of experiencing VI (OR = 0.33; 95% CI: 0.25–0.43) compared to those aged 6–17. Additionally, women were found to have 1.58 times higher odds (95% CI: 1.22–2.10) of VI compared to men. Participants who had eye examinations for one year or more exhibited 3.64 times higher odds (95% CI: 2.70–4.92) of VI compared to those who never had their eyes examined before. Furthermore, participants with uncorrected RE had lower odds of experiencing VI compared to emmetropic participants (OR = 0.15; 95% CI: 0.10–0.22), as shown in Table 4.

### 3.6. The Most Common Cause of Visual Impairment

The results showed that the primary cause of VI was uncorrected RE (81.4%), followed by amblyopia (13.5%) and cataract (3.2%), as illustrated in Table 5.

## 4. Discussion

This study was carried out in Duhknah, located within the Qassim Province of Saudi Arabia, with the primary aim of assessing the prevalence of visual impairment (VI) and refractive errors, as well as identifying their underlying causes and associated risk factors among the local residents. The results revealed that 72.2% of participants had never undergone an eye examination before, highlighting a significant gap in access to basic eye care services. Furthermore, 39.8% of the individuals were found to have some form of VI, with 4.1% classified as having severe VI and 3.1% considered as blind. The most common cause of VI among participants was uncorrected refractive errors, accounting for 81.4% of cases. This was followed by amblyopia at 13.5%, and cataracts at 3.2%.

In the present study, the prevalence of VI was found to be 39.8%, representing the highest rate reported among studies conducted in rural areas of Saudi Arabia [5,6,7]. Previous research in the different regions of Saudi Arabia have documented prevalence rates of VI ranging from 13.9% to 32.1%. When compared to global data, the prevalence of VI in Saudi Arabia appears notably higher [10,11,12,13]. For instance, a comprehensive Chinese systematic review reported a prevalence of 10.9% for VI [11], while another study encompassing data from 13 different regions in China revealed prevalence rates between 5.02% and 2.44% [10]. In the United States, the prevalence of VI varies considerably, ranging from 1.6% to 24.8% among individuals under 65 years old, and between 2.2% and 26.6% in populations aged 65 and above [12]. A previous study estimated that visual impairment results in an annual global productivity loss of approximately USD410.7 billion [14]. The elevated prevalence observed in Saudi Arabia underscores a critical public health concern, emphasizing the need to enhance access to comprehensive eye care services and general healthcare infrastructure. Addressing these issues is essential to align with Saudi Arabia’s Vision 2030 goals, where one of it aims is to improve the health sector and overall health of Saudi Arabia’s citizens [15].

Regarding the primary causes of VI identified in this study, uncorrected RE were responsible for 81.4% of cases, followed by amblyopia at 13.5%, and cataracts at 3.2%. These findings are consistent with other studies within Saudi Arabia, where uncorrected RE remain the predominant cause of VI [5,6,7]. However, on a global scale, the most common cause of VI is typically cataracts, followed by uncorrected REs [10,11,14]. The trend in Saudi Arabia, where uncorrected REs are the most cause of VI, likely reflects better access to cataract surgery and public health efforts targeting cataracts. Meanwhile, limited use of corrective lenses and lifestyle changes, such as increased screen time and lack of outdoor activities, may contribute to the higher prevalence of RE. Globally, cataracts remain more common due to limited surgical access in many developing nations.

Furthermore, the present study found that the risk of visual impairment was significantly lower among older participants compared to younger ones (*p* < 0.001) [7,11,16]. In the current study, women had approximately 1.58 times higher odds of experiencing VI. This gender disparity highlights the need for gender-sensitive approaches in eye health programs to ensure equitable access and treatment. Previous study has shown that women are disproportionately affected by vision loss due to a combination of biological, behavioral, and social factors. For example, women tend to live longer than men, increasing their exposure to age-related eye diseases such as cataracts and macular degeneration [17]. Furthermore, gender-based barriers such as limited access to eye care services, cultural norms, and socioeconomic disadvantages contribute to reduced utilization of eye health services among women [3,17]. These findings underscore the importance of designing eye health interventions that address the unique needs and challenges faced by women.

Amblyopia was identified as the second most common cause of VI in this study, accounting for 13.5% of cases. This figure is relatively high compared to other reports; for example, a study conducted in the Qassim Province of Saudi Arabia found amblyopia responsible for 8.8% of causes of VI [7]. On a global scale, the prevalence of amblyopia among children is estimated to be around 1.36% [18], indicating a significantly higher burden within the studied population [18]. The higher rate of amblyopia-related visual impairment in Qassim region may be due to limited early screening, delayed treatment, and lower public awareness compared to global settings. Furthermore, differences in study methods and access to pediatric eye care may also contribute to this variation.

Cataract was the third most common cause of VI in the current study, contributing to 3.2% of the cases. This aligns closely with the global cataract related VI prevalence of 3.9% [18]. Both amblyopia and cataracts are major yet reversible causes of VI. Despite their treatable nature, they can impose substantial burdens, not only on an individual’s quality of life, but also on the broader economic productivity and healthcare resources of affected nations [18,19,20,21,22,23,24]. For example, in 2017, the economic burden of vision loss was estimated at USD134.2 billion, including USD98.7 billion in direct costs and USD35.5 billion in indirect costs, along with significant productivity losses [25]. Amblyopia, if untreated, leads to lifelong productivity loss and reduced quality of life. Globally, the burden is even greater in low- and middle-income countries due to limited access to eye care services. These findings highlight the need for early intervention to reduce healthcare costs and improve outcomes.

Amblyopia, often beginning in childhood, can lead to lifelong VI if not detected and treated early. As it typically affects individuals during their formative and most productive years, its impact extends beyond health, influencing educational performance, employment opportunities, and overall social participation [18,26,27,28]. This underlines the importance of routine vision screening in children and timely intervention, which can successfully reverse the condition in most cases. Cataract, on the other hand, is an age-related condition that, while not preventable, can be effectively managed through surgical treatment [23]. Early diagnosis and access to cataract surgery can significantly reduce the risk of long-term VI, preserving independence and reducing care costs among the elderly population.

The findings of this study emphasize the importance of addressing the challenges of accessing healthcare in rural areas, with almost 72.2% of study participants who lived in Duhknah having never undergone an eye examination before, and the closest eye care facility is located 133 km from Duhknah. Considering ongoing health reforms in Saudi Arabia and the targets set for vision 2030, such studies call attention to urgent health issues and may inform efforts for constructive progress in health accessibility as well as improve the quality of life. Increasing accessibility to eye care services is essential to combat the high burden of VI in rural areas [15]. Early detection and treatment of VI can be achieved by mobile eye clinics, as well as community outreach or school-screening programs. Community eye health campaigns can increase awareness and eliminate avoidable blindness, especially among high-risk groups such as older adults, women, and individuals with systemic diseases. Policymakers should focus on subsidizing eye care health and encourage partnerships and collaborations between the public and private sectors to enhance accessibility and affordability of services. In addition, this study suggests collaborating with local eye care providers and NGOs in Saudi Arabia to address the challenges in delivering eye care services and developing sustainable public health interventions. Further research is also required to assess the cost-effectiveness of such interventions in rural areas. Consequently, this would contribute positively to creating well-organized strategy plans aimed at decreasing the prevalence of VI and enhancing eye care services in the most underserved communities.

Despite the significance of the findings, several limitations should be acknowledged. First, cycloplegic refraction could not be performed for the pediatric age group, which may have affected the accuracy of refractive error diagnoses in children [29]. Second, the screening process was not randomized, as it was conducted as part of a community health campaign. This means that individuals voluntarily presented themselves for eye examinations, potentially skewing the sample toward those already experiencing visual problems. Consequently, individuals without apparent symptoms may have been underrepresented, leading to a possible underestimation of the prevalence of VI. Nevertheless, despite these limitations, the present study provides valuable insights and highlights key figures that underscore the importance of strengthening primary care services, particularly in underserved rural communities.

## 5. Conclusions

This study revealed a high prevalence of VI in a rural community in Qassim Province, with uncorrected RE as the predominant cause. The results showed a significant proportion of participants (72.2%) had never undergone an eye examination before. The overall prevalence of RE among participants was 70.7% and only 13% of them were using glasses. The elevated rates of VI, particularly among women and older adults, point to major gaps in eye care accessibility and awareness. These findings underscore a critical public health issue: the lack of accessible and primary care services in rural areas. The data strongly suggests the need for community-based initiatives and public health programs focused on early detection, treatment, and prevention of avoidable VI, especially through mobile eye screening and awareness campaigns. Addressing these gaps is vital for improving individual well-being and achieving broader healthcare goals in Saudi Arabia’s Vision 2030.

## Figures and Tables

**Table 1:** Demographic characteristics and visual impairment status of participants.

Demographic Characteristics	Visual Impairment n (%)	Total n (%)	Chi-Square Tests *p*-Value
Age Group (Years)			
6–10	121 (30.2)	401 (43.2)	0.000
11–17	45 (27.8)	162 (17.4)	
18–39	104 (53.1)	196 (21.1)	
40–49	35 (49.3)	71 (7.6)	
50–59	36 (65.5)	55 (5.9)	
60 and above	30 (68.2)	44 (4.7)	
Gender			0.001
Male	169 (34.7)	487 (52.4)	
Females	202 (45.7)	442 (47.6)	
Last Examination Date			
Never	210 (31.3)	671 (72.2)	0.000
One year or more	161 (62.4)	258 (27.8)	
Eyeglass Usage			
Yes	95 (78.5)	121 (13.0)	0.000
No	276 (34.2)	808 (87.0)	
Total	371 (39.8) [95%CI, 36.0–44.2]	929 (100)	

**Table 2:** Prevalence of presenting visual impairment by gender and age groups.

Presenting Visual Impairment						Total
Characteristics	Mild VI n (%)	Moderate VI n (%)	Severe VI n (%)	Blindness n (%)	NAD n (%)	
Age (years)	Chi-Square Tests, *p* < 0.001					
6–10	80 (20.0)	25 (6.2)	7 (1.7)	10 (2.5)	179 (69.6)	401
11–17	20 (12.3)	10 (6.2)	5 (3.1)	10 (6.2)	117 (72.2)	162
18–39	57 (29.1)	32 (16.3)	11 (5.6)	2.0%	92 (46.9)	196
40–49	19 (26.8)	11 (15.5)	5 (7.0)	0 (0.0)	36 (50.7)	71
50–59	12 (21.8)	15 (27.3)	5 (9.1)	2 (3.6)	21 (38.2)	55
60 and above	13 (29.5)	9 (20.5)	5 (11.4)	3 (6.8)	14 (31.8)	44
Gender	Chi-Square Tests, *p* = 0.015					
Male	91 (18.7)	45 (9.2)	17 (3.5)	16 (3.3)	318 (65.3)	487
Female	110 (24.9)	57 (12.9)	21 (4.8)	13 (2.9)	241 (54.5)	442
Total	201 (21.6)	102 (11.0)	38 (4.1)	29 (3.1)	559 (60.2)	929
95% CI	21.6, 18.9–24.9	11.0, 8.9–13.3	4.1, 2.9–5.6	3.1, 2.1–4.5	60.2, 55.3–65.4	

VI = Visual impairment, NAD = No abnormality detected.

**Table 3:** Prevalence of refractive error by age groups and gender.

Refractive Error 657 (70.7% [95% CI, 65.4–76.3])					
Characteristics	Emmetropia n (%)	Hyperopia n (%)	Myopia n (%)	Astigmatism n (%)	Total
Age (years)	Chi-Square Tests, *p* < 0.001				
6–10	140 (34.9)	107 (26.7)	73 (18.2)	81 (20.2)	401
11–17	36 (22.2)	20 (12.3)	91 (56.2)	15 (9.3)	162
18–39	60 (30.6)	9 (4.6)	118 (60.2)	9 (4.6)	196
40–49	21 (29.6)	10 (14.1)	36 (50.7)	4 (5.6)	71
50–59	10 (18.2)	24 (43.6)	12 (21.8)	9 (16.4)	55
60 and above	5 (11.4)	21 (47.7)	13 (29.5)	5 (11.4)	44
Gender	Chi-Square Tests, *p* = 0.025				
Male	142 (29.2)	93 (19.1)	183 (37.6)	69 (14.2)	487
Female	130 (29.4)	98 (22.2)	160 (36.2)	54 (12.2)	442
Total	272 (29.3)	191 (20.6)	343 (36.9)	123 (13.2)	929
95% CI	29.3, 25.9–32.9	20.6, 17.8–23.7	36.9, 33.1–41.0	13.2, 11.0–15.8	

**Table 4:** Multinomial logistic regression analysis of the impact of demographic characteristics and refractive error on visual impairment.

Characteristics	Adjusted Odds Ratio (95% CI)	*p*-Value
Age group (years)		
6–17	Reference	
18–90	0.33 (0.25–0.43)	<0.001
Gender		
Male	Reference	
Female	1.58 (1.22–2.10)	0.001
Date of Last Examination		
Never	Reference	
One year or more	3.64 (2.70–4.92)	<0.001
Refractive Error (RE)		
Emmetropia	Reference	
Uncorrected RE	0.15 (0.10–0.22)	<0.001

**Table 5:** The most common causes of visual impairment.

Causes	Presenting VI				
	Mild VI n (%)	Moderate VI n (%)	Severe VI n (%)	Blindness n (%)	Total n (%)
Refractive error	174 (86.6)	79 (77.5)	27 (71.1)	17 (58.6)	301 (81.4)
Amblyopia	19 (9.5)	16 (15.7)	6 (15.8)	8 (10.3)	50 (13.5)
Cataract	2 (1.0)	2 (2.0)	3 (7.9)	3 (5.4%)	12 (3.2)
Diabetic retinopathy	2 (1.0)	2 (2.0)	1 (2.6)	1 (3.4)	6 (1.6)
Congenital disorders	4 (2.0)	3 (2.9)	1 (2.6)	0 (0.0)	8 (2.2)
Total	201 (21.6)	102 (11.0)	38 (4.1)	29 (3.1)	370 (39.8)

## Data Availability

There are no additional data deposited on any other site other than in this manuscript.

## Abbreviations

The following abbreviations are used in this manuscript:

ICD-11	International Classification of Diseases 11th revision
VI	Visual impairment
VA	Visual acuity
RE	Refractive error

## References

[1] GBD 2019 Blindness and Vision Impairment Collaborators, Vision Loss Expert Group of the Global Burden of Disease Study (2021). Trends in prevalence of blindness and distance and near vision impairment over 30 years: An analysis for the Global Burden of Disease Study. Lancet Glob. Health.

[2] Alrasheed S. (2023). Systematic review and meta-analysis of childhood visual impairment in the Eastern Mediterranean Region. East. Mediterr. Health J..

[3] Alrasheed S.H. (2021). A systemic review of barriers to accessing paediatric eye care services in African countries. Afr. Health Sci..

[4] Alrasheed S.H., Alghamdi W.M. (2022). Parents’ Awareness of and Perspectives on Childhood Refractive Error and Spectacle Wear in Saudi Arabia. Sultan Qaboos Univ. Med. J..

[5] Alghamdi W., Ovenseri Ogbomo G.O. (2021). The prevalence and causes of visual impairment in Dariyah, a rural community in Saudi Arabia. Afr. Vis. Eye Health.

[6] Al-Shaaln F.F., Bakrman M.A., Ibrahim A.M., Aljoudi A.S. (2011). Prevalence and causes of visual impairment among Saudi adults attending primary health care centers in northern Saudi Arabia. Ann. Saudi Med..

[7] Aldakhil S., Alrasheed S.H., Mutwaly R.F., Alenezi B., Alrabiah S., Alnawmasi M.M., Almutairi N.M., Alhoshan S.A., Alnasser B.N. (2025). Prevalence, Causes, and Risk Factors Associated with Visual Impairment in Qbah, a Rural Community in the Qassim Region of Saudi Arabia. Healthcare.

[8] Abdi Ahmed Z., Alrasheed S.H., Alghamdi W. (2020). Prevalence of refractive error and visual impairment among school-age children of Hargesia, Somaliland, Somalia. East. Mediterr. Health J..

[9] Harrison J.E., Weber S., Jakob R., Chute C.G. (2021). ICD-11: An international classification of diseases for the twenty-first century. BMC Med. Inform. Decis. Mak..

[10] Cao K., Hu A.L., Zhou X.Y., Zhuang W.J., Hu Z.L., Zhang M.L., Huang W.Y., Liang X.L., Liu Q.H., Song Z.M. (2025). A cross-sectional survey of blindness and visual impairment among adults in 13 regions of China from 2021 to 2022. Chin. J. Ophthalmol..

[11] Zou M., Guo D., Chen A., Young C.A., Li Y., Zheng D., Jin G. (2021). Prevalence of visual impairment among older Chinese population: A systematic review and meta-analysis. J. Glob. Health.

[12] Rein D.B., Lamuda P.A., Wittenborn J.S., Okeke N., Davidson C.E., Swenor B.K., Saaddine J., Lundeen E.A. (2021). Vision Impairment and Blindness Prevalence in the United States: Variability of Vision Health Responses across Multiple National Surveys. Ophthalmology.

[13] Alrasheed S.H., Naidoo K.S., Clarke-Farr P.C. (2016). Prevalence of visual impairment and refractive error in school-aged children in South Darfur State of Sudan. Afr. Vis. Eye Health.

[14] Burton M.J., Ramke J., Marques A.P., Bourne R.R., Congdon N., Jones I., Tong B.A.A., Arunga S., Bachani D., Bascaran C. (2021). The Lancet Global Health Commission on global eye health: Vision beyond 2020. Lancet Global Health.

[15] Alasiri A.A., Mohammed V. (2022). Healthcare Transformation in Saudi Arabia: An Overview Since the Launch of Vision 2030. Health Serv. Insights.

[16] Xu L., Cui T., Yang H., Hu A., Ma K., Zheng Y., Sun B., Li J., Fan G., Jonas J.B. (2006). Prevalence of visual impairment among adults in China: The Beijing Eye Study. Am. J. Ophthalmol..

[17] Aninye I.O., Digre K., Hartnett M.E., Baldonado K., Shriver E.M., Periman L.M., Grutzmacher J., Clayton J.A., Society for Women’s Health Research Women’s Eye Health Working Group (2021). The roles of sex and gender in women’s eye health disparities in the United States. Biol. Sex Differ..

[18] Hu B., Liu Z., Zhao J., Zeng L., Hao G., Shui D., Mao K. (2022). The Global Prevalence of Amblyopia in Children: A Systematic Review and Meta-Analysis. Front. Pediatr..

[19] Aljohani S., Aldakhil S., Alrasheed S.H., Tan Q.Q., Alshammeri S. (2022). The Clinical Characteristics of Amblyopia in Children Under 17 Years of Age in Qassim Region, Saudi Arabia. Clin. Ophthalmol..

[20] Hashemi H., Pakzad R., Yekta A., Aghamirsalim M., Pakbin M., Ramin S., Khabazkhoob M. (2020). Global and regional prevalence of age-related cataract: A comprehensive systematic review and meta-analysis. Eye.

[21] Cedrone C., Culasso F., Cesareo M., Mancino R., Ricci F., Cupo G., Cerulli L. (1999). Prevalence and incidence of age-related cataract in a population sample from Priverno, Italy. Ophthalmic Epidemiol..

[22] Li T., He T., Tan X., Yang S., Li J., Peng Z., Li H., Song X., Wu Q., Yang F. (2009). Prevalence of age-related cataract in high-selenium areas of China. Biol. Trace Elem. Res..

[23] Nam G.E., Han K., Ha S.G., Han B.D., Kim D.H., Kim Y.H., Cho K.H., Park Y.G., Ko B.J. (2015). Relationship between socioeconomic and lifestyle factors and cataracts in Koreans: The Korea National Health and Nutrition Examination Survey 2008-2011. Eye.

[24] Mangione C.M., Phillips R.S., Lawrence M.G., Seddon J.M., Orav E.J., Goldman L. (1994). Improved visual function and attenuation of declines in health-related quality of life after cataract extraction. Arch. Ophthalmol..

[25] Rein D.B., Wittenborn J.S., Zhang P., Sublett F., Lamuda P.A., Lundeen E.A., Saaddine J. (2022). The Economic Burden of Vision Loss and Blindness in the United States. Ophthalmology.

[26] Fu Z., Hong H., Su Z., Lou B., Pan C.W., Liu H. (2020). Global prevalence of amblyopia and disease burden projections through 2040: A systematic review and meta-analysis. Br. J. Ophthalmol..

[27] Alrasheed S.H., Elmadina A.E.M. (2020). Effect of Binocular Vision Problems on Childhood Academic Performance and Teachers’ Perspective. Pak. J. Ophthalmol..

[28] Alghamdi W.M., Alluwimi M.S., Aldakhil S.A., Moafa M.A., Alghamdi M.A. (2022). Refractive errors and binocular vision anomalies among young university students. Glob. J. Health Sci..

[29] Zhu D., Wang Y., Yang X., Yang D., Guo K., Guo Y., Jing X., Pan C.W. (2016). Pre- and Postcycloplegic Refractions in Children and Adolescents. PLoS ONE.

